# A System Based-Approach to Examine Cytokine Response in Poxvirus-Infected Macrophages

**DOI:** 10.3390/v10120692

**Published:** 2018-12-05

**Authors:** Pui-San Wong, Richard Sutejo, Hui Chen, Sock-Hoon Ng, Richard J. Sugrue, Boon-Huan Tan

**Affiliations:** 1 Defence Medical and Environmental Research Institute, DSO National Labs, Singapore 117510, Singapore; wpuisan@dso.org.sg (P.-S.W.); ng_sock_hoon@dso.org.sg (S.-H.N.); 2 School of Biological Sciences, Nanyang Technological University, Singapore 637551, Singapore; richard.sutejo@13l.ac.id (R.S.); chenh1@gis.a-star.edu.sg (H.C.); rjsugrue@ntu.edu.sg (R.J.S.); 3 Infection and Immunity, LKC School of Medicine, Nanyang Technological University, Singapore 308232, Singapore

**Keywords:** poxvirus, macrophages, RAW cells, pro-inflammatory cytokines

## Abstract

The poxviruses are large, linear, double-stranded DNA viruses about 130 to 230 kbp, that have an animal origin and evolved to infect a wide host range. Variola virus (VARV), the causative agent of smallpox, is a poxvirus that infects only humans, but other poxviruses such as monkey poxvirus and cowpox virus (CPXV) have crossed over from animals to infect humans. Therefore understanding the biology of poxviruses can devise antiviral strategies to prevent these human infections. In this study we used a system-based approach to examine the host responses to three orthopoxviruses, CPXV, vaccinia virus (VACV), and ectromelia virus (ECTV) in the murine macrophage RAW 264.7 cell line. Overall, we observed a significant down-regulation of gene expressions for pro-inflammatory cytokines, chemokines, and related receptors. There were also common and virus-specific changes in the immune-regulated gene expressions for each poxvirus-infected RAW cells. Collectively our results showed that the murine macrophage RAW 264.7 cell line is a suitable cell-based model system to study poxvirus host response.

## 1. Introduction

The poxviruses are a family of linear, double-stranded DNA (dsDNA) viruses about 130 to 230 kbp that belong to the family *Poxviridae* [1]. They are characterised by having large and complex genomes which can encode for an array of different proteins. These virus-encoded proteins play an important part at different stages of the virus replication cycle, such as DNA genome replication and overcoming the host responses to infection. Different members of the poxvirus family have evolved to infect an array of different animals. In this context the *Variola* virus (VARV), the causative agent of smallpox, has evolved to infect humans, and humans are the only known natural host for VARV. The VARV is the causative agent of smallpox, and given its limited host range, infection in the natural environment was eradicated by mass vaccination programs organized by the World Health Organization. Although naturally occurring smallpox infection has been eradicated, stockpiles of the VARV are maintained in secured laboratories, but the possibility of accidental or intentional release of VARV into the natural environment remains. In addition, a wide variety of animals can be infected by different members of the poxvirus family, and the transfer of poxviruses across host-species boundaries from animals to humans is a possibility. In this context the exact origin of the VARV is uncertain, but an animal origin of the VARV is suggested (reviewed in [2,3]). The innate immune response in the new host is likely to be an important barrier to infection by a poxvirus originating in another animal host. Therefore for a zoonosis involving a poxvirus to occur, the new virus would have to overcome the immunological defenses in the new host, in particular the host innate immune responses. Understanding the biology of VARV and other members of the family *Poxviridae* are of continued interest.

There are technical and logistical issues to examining smallpox virus infection in either cell-based or animal-based models of infection. These studies require high-containment facilities to handle the VARV, which are both costly to maintain and are inconvenient to use. Therefore it is desirable to use surrogate poxviruses that can be handled at Biosafety level 2 and which can be used to establish disease parameters prior to using VARV in high-containment. In animal models that were used to study VARV infection the role of macrophages in virus dissemination in the host (via viremia) was proposed [4,5]. In early studies it was demonstrated that in mice, macrophages are crucial to control the infection by the murine poxvirus ectromelia virus (ECTV) [6]. ECTV can replicate in macrophage cells and infected macrophages can directly contribute to virus dissemination within the host [7].

Given the importance of macrophages in the pathology of poxvirus infection we have performed a system-based approach to examine the effect of poxvirus infection on the host cell transcriptome and the pro-inflammatory responses. Poxviruses are well-known for their ability to evade host responses during viral infection by secreting virus-encoded homologues of host cytokines, chemokines and their receptors (review by [8]). In this study we examined the host responses to three different Orthopoxviruses in the murine macrophage RAW 264.7 cell line. This is an established cell model system to investigate macrophage infection for ECTV, myxoma virus (a poxvirus from different genus, Leporipoxvirus) and other DNA viruses such as Herpes simplex virus type-I, murine cytomegalovirus, and adenoviruses [9,10,11,12,13,14]. The ECTV naturally infects mice and in this study infection with ECTV Moscow strain was performed in parallel with the cowpox virus (CPXV) Brighton strain, and vaccinia virus (VACV) Lister strain. Both the CPXV and VACV can infect human host and share a high genome similarity when aligned with the smallpox virus genome, suggesting that both can be used as suitable surrogates to examine smallpox infection [15,16].

## 2. Materials and Methods

### 2.1. Viruses, Cells, Antibodies

All the viruses and cell lines for this study were acquired from American Type Culture Collection (ATCC), USA. The CPXV Brighton strain (VR-302); VACV Elstree (Lister) strain (VR-1549); and ECTV Moscow strain (VR-1374) were used in this study. Baby hamster kidney (BHK-21) cells (CCL-10), African green monkey Kidney (Vero) cells (CCL-81) and human lung epithelial carcinoma (A549) cells (CCL-185) were maintained in Dulbecco’s Modified Eagle’s Medium (DMEM) (Invitrogen, USA) containing 10% fetal bovine serum (FBS) (Invitrogen, Carlsbad, CA, USA) and 1% penicillin/streptomycin (Invitrogen, Carlsbad, CA, USA). RAW 264.7 cells (TIB-71) were maintained in Minimal Essential Medium (MEM), containing 10% FBS and 1% penicillin/streptomycin (Invitrogen, Carlsbad, CA, USA). BHK-21 cells were used to propagate the poxviruses and Vero cells for virus titration. Infections with BHK-21 and Vero cells were carried out in DMEM and in 2% FBS, at 37 °C in the presence of 5% CO_2_. The infection of RAW cells with each poxvirus was carried out in MEM and 2% FBS, also at 37 °C and in the presence of 5% CO_2._ The anti-VACV (8101) (Virostat, Augusta, ME, USA) was a rabbit polyclonal antibody raised against VACV but was reactive to poxviruses, including CPXV and ECTV, and anti-mouse IgG conjugated to Fluorescein (FITC) was purchased from Chemicon (Waltham, MA, USA).

### 2.2. Virus Purification and Plaque Assay

BHK-21 cells were infected with each poxvirus at 95% confluence at a multiplicity of infection (MOI) = 10 and incubated for 1 h in DMEM with 2% FBS at 37 °C, in the presence of 5% CO_2_. At 48 h post-infection (hpi), the infected cells were scraped down with the tissue culture supernatant in 10mM Tris pH9.0. Sterile 3 mm glass beads (Sigma-Aldrich, St. Louis, MO, USA) were added to the infected cell suspension, vortexed at a high speed for 5 s and sonicated (Jac Ultrasonic 1505 waterbath, Korea) for approximately 30 s. The infected cell suspension, together with the glass beads, was centrifuged at 1200× *g* for 10 min at 4 °C. The clarified supernatant was transferred to a clean tube. An additional 10 mL of 10 mM Tris pH 9.0 was added to the glass beads and a second round of vortexing, sonication and centrifugation was repeated. Both clarified supernatants were pooled, applied to a 40% *w/v* sucrose cushion and centrifuged at 130,000× *g* in a Beckman SW28 rotor for 2 h at 4 °C. The virus pellets were resuspended in 10 mM Tris solution pH 9.0, sonicated briefly and applied onto a gradient containing 25% to 45% *w/v* sucrose, prepared in 10 mM Tris pH 9.0. The virus suspension was subjected to ultracentrifugation at 120,000× *g* in a Beckman SW41 rotor for 1 h at 4 °C. A band observed at the 40–45% interface was collected in 10 mM Tris solution, pH 9.0. For plaque titration, Vero cells were seeded at 1 × 10^5^ cells per well in 24-well plate and when near confluence, inoculated with serial dilutions of each poxvirus. At 48 hpi, the infected cells were monitored for plaques and stained with 0.5% crystal violet in 25% formaldehyde when visible plaques were observed.

### 2.3. Examination by Immunoflorescence Microscopy

RAW cells were seeded onto 13 mm glass coverslips and infected with each poxvirus at a MOI = 10. At the respective time point of infection, the cells were fixed with 4% paraformaldehyde (Sigma-Aldrich, St. Louis, MO, USA) and permeabilized in 0.1% saponin (Sigma-Aldrich, St. Louis, MO, USA). The cells were labelled with 1:100 dilution of Anti-VACV and next, anti-mouse IgG conjugated to FITC. The stained cells were mounted onto slides using Dakocytomation (Dako, Palo Alto, CA, USA) and visualized using florescence microscope (Model BX51, Olympus, Tokyo, Japan).

### 2.4. Quantitative PCR to Measure the virus Replication Kinetics

RAW cells were infected with each poxvirus at a MOI = 10. The poxvirus-infected cells were harvested at 2, 10, 24 and 48 hpi by scraping the infected cells in 1× phosphate buffered solution (PBS) (Invitrogen, Carlsbad, CA, USA), and pelleting the cells at 4 °C. Total DNA was extracted from each infected cell pellet using the Qiagen DNA minikit (Qiagen, Hilden, Germany) according to manufacturer’s instructions and the viral genome copy measured using real-time PCR reported in [17]. Standard curves were generated based on the plasmid DNA controls as prepared in [17]. At each time point, the relative fold-change (FC) for each virus-infected RAW cells was normalized with mock-infected RAW cells, and the genome copy number calculated based on the standard curves. The qPCR was conducted in duplicate, and the data presented are the average from 2 independent experiments.

### 2.5. Microarray Experiment and Data Analysis

RAW cells were either mock-infected or infected with each purified poxvirus at a MOI = 10. At 2 and 16 hpi, the cells were harvested at 4 °C using RNAlater (Ambion, Waltham, MA, USA) diluted in 1:1 with PBS buffer, aliquoted, pelleted and stored at −80 °C. Total RNA was extracted using RNeasy mini kit (Qiagen, Hilden, Germany) and quantified using the Nanodrop ND-1000 Spectrophotometer (Thermo Fischer Scientific, Waltham, MA, USA). Double-stranded cDNA was synthesized from 3 µg of total RNA with the GeneChip One-cycle cDNA synthesis kit (Affymetrix, Santa Clara, CA, USA), followed by synthesis of biotin-labelled cRNA using the GeneChip IVT labelling kit (Affymetrix, Santa Clara, CA, USA), according to manufacturer’s instructions. After cRNA fragmentation, 15 µg of biotin-labelled cRNA was hybridised to the GeneChip mouse genome 430 2.0 Array (Affymetric, Santa Clara, CA, USA). Three independent experiments were performed and the data analyzed separately. The arrays were washed and stained using the Hybridisation, Wash and Stain Kit (Affymetric, Santa Clara, CA, USA) and the GeneChip Fluidics Station 450 (Affymetrix, Santa Clara, CA, USA) according to standard Affymetrix protocols. Finally the arrays were scanned with the GeneChip scanner 3000 (Affymetrix, Santa Clara, CA, USA). Quality control, GeneChip hybridization and data acquisition were performed according to the standard protocols available from Affymetrix. Affymetrix CHP files were generated from GeneChip Operating Software (GCOS) version 1.1 (Affymetrix, Santa Clara, CA, USA) and subsequently imported into GeneSpring 11.0 (Agilent, Santa Clara, CA, USA) for analysis. The data was normalized using external genes control or spike-in normalization, which includes *dap*, *lys*, *phe,* and *thr* for poly-A controls to normalize cRNA synthesis variability, and *bioB*, *bioC*, *bioD,* and *cre* for hybridization controls to normalize chip hybridization variability, as recommended by Affymetrix. After the signal intensity of each chip was normalized, the data sets were normalized to mock to obtain the FC value. The genes from the data set were considered significantly changed if the gene expression showed more than 2-FCs with the adjusted *p*-value < 0.05, using Benjamini–Hochberg False Discovery Rate. The gene changes are represented by the number of probe-sets showing more than 2-FCs. To identify significantly changed probe-sets that are common to 2 or 3 poxviruses, the % value is expressed with the number of probe-sets identified in common between the 2 or 3 viruses/total number of probe-sets identified for each of the 2 or 3 viruses. The known pathways of differentially expressed genes associated with metabolism and signaling were investigated by canonical pathway analysis using Ingenuity Pathways analysis (IPA; Ingenuity Systems http://www.ingenuity.com). Those differentially expressed genes with known IDs and corresponding expression FCs were uploaded into the software. *p*-value was used to determine the probability of association between the genes in the dataset and the canonical pathway. IPA uses a right-tailed Fisher’s exact test to calculate p-value for canonical pathway analysis, with a *p*-value cut-off of ≤ 0.05. All microarray data was deposited as MIAME-compliant data submissions with accession number GSE120616 in the Gene Expression Omnibus.

### 2.6. PCR Arrays

Total RNA was extracted from poxvirus-infected RAW cells as described above. First strand cDNA was reverse-transcribed from 1 µg of total RNA using the RT^2^ First Strand Kit (SABiosciences, Hilden, Germany) according to the manufacturer’s protocols. The cDNA was pre-mixed with RT^2^ Real-TimeTM SYBR Green/Fluorescein PCR Master Mix, and applied on two types of array: RT^2^ Profiler PCR Array Mouse Inflammatory Cytokines and Receptors plates, RT^2^ Profiler PCR Array Mouse Interferon and Receptors plates (SABiosciences, Hilden, Germany). Quantitative PCR was carried out on the Applied Biosystems 7500 Fast Real-Time PCR system (Applied Biosystems, Foster City, CA, USA) following the protocols and settings from the manufacturer. The FC was calculated by 2-CT method with the software available at http://www.sabiosciences.com/pcrarraydataanalysis.php and normalized with the housekeeping genes. The student’s t-test was used to calculate statistically significant FC for each cytokine at each time point, with a cut-off for FC set to ≥ 3. Two independent experiments were performed and measured separately.

### 2.7. Cytokine Assay

Supernatant from poxvirus infected-RAW cells were centrifuged at 10,000× *g* for 10 min at 4 °C after which the supernatant was analyzed on the Bio-Plex Protein Array System (BioRad) using the Bio-Plex Mouse Cytokine 23-Plex Panel (#M60009RDPD, Bio-Rad, Inc., Hercules, CA, USA) according to manufacturer’s instructions. Data were analyzed using the Bio-Plex Manager software (Bio-Rad, Inc., Hercules, CA, USA) according to manufacturer’s instructions. One-way analysis of variance (ANOVA) was conducted with Tukey’s post-tests and two-way ANOVA with Bonferroni’s post-tests. *p*-values < 0.05 were considered as statistically significant.

## 3. Results

### 3.1. RAW Cells Were Susceptible to Infection by CPXV, ECTV and VACV

**Figure 1:**
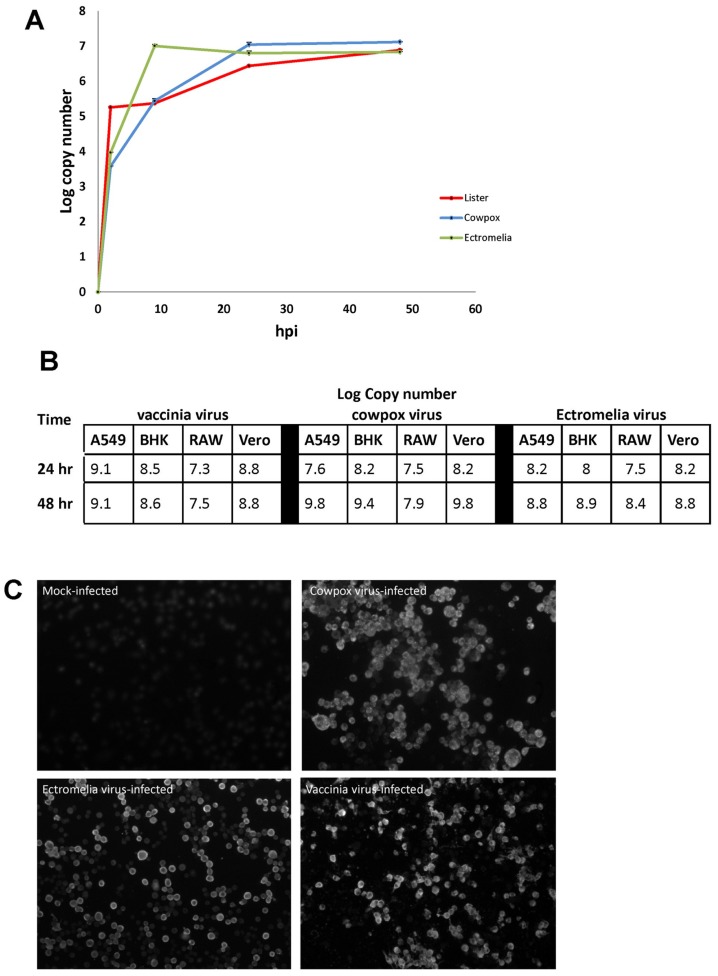
Examination of RAW cells infected with cowpox virus (CPXV), ectromelia virus (ECTV) and vaccinia virus (VACV) using quantitative PCR (qPCR) to measure the virus replication kinetics and immunofluorescence microscopy to measure infectivity. (**A**) RAW cells were infected with each poxvirus and the virus replication kinetics measured at 2, 10, 24 and 48 h post-infection (hpi) with qPCR. The growth curve is plotted with viral genome copies as log copy number on the y-axis and time of infection as hpi on the x-axis (♦ represent VACV; ■ represents CPXV; ▲ represents ECTV). (**B**) A549, BHK, RAW and VERO cells were infected with each poxvirus and the virus replication kinetics measured at 24 and 48 hpi with qPCR. (**C**) RAW cells were mock-infected and infected with each poxvirus and at 16 hpi, stained with anti-VACV and examined using immunoflorescence microscopy (objective ×20).

RAW cells were infected respectively with CPXV, ECTV and VACV using a MOI = 10 plaque-forming units (PFU). At 2, 10, 24 and 48 hpi, total DNA were extracted from the poxvirus-infected cells and the viral genome quantitated by PCR as described in (17). All 3 poxviruses displayed similar virus replication kinetics in RAW cells and at between 2 and 24 hpi a 500–1000-fold increase in the virus genomes levels was recorded (Figure 1A). At between 24 and 48 hpi only a small increase in virus genome copy number was noted, indicating a plateau in the increase in virus genome copy levels during this time interval. At 24 and 48 hpi, CPXV was titered at 1.7 × 10^5^ pfu/mL and 6 × 10^6^ pfu/mL respectively, and that for VACV at 6.5 × 10^4^ pfu/mL and 1 × 10^6^ pfu/mL/. This increase in virus titer for both CPXV and VACV is consistent with the increase in virus genome copy number. We also used qPCR to measure the virus load in RAW cells with that in A459, BHK and VERO cells. In general each of the poxviruses used in this study appeared to replicate better in these other cell lines than in RAW cells (Figure 1B). The three viruses exhibited a slight difference in the peak levels of virus replication in RAW cells at 24 hpi. RAW cells were infected with CPXV, ECTV, and VACV, and at 16 hpi, the poxvirus-infected cells were stained with anti-VACV and examined by immunoflorescence (IF) microscopy (Figure 1C). The anti-VACV is a rabbit polyclonal antibody raised against VACV but is cross-reactive with CPXV and ECTV. In each case we noted that greater than 90% of the cells exhibited anti-VACV staining. CPXV-infected RAW cells displayed speckled staining in the cytoplasm whereas the ECTV-infected RAW cells displayed peripheral staining at the cytoplasm, and both patterns were observed in the VACV-infected RAW cells. The poxvirus-infected cells were resin-embedded, thin-sectioned and the thin-sections visualized by transmission electron microscopy (TEM) (Figure S1E). In both the CPXV and VACV-infected RAW cells, intracellular mature virus particles were visibly observed in the cytoplasm of the cells. The boxed area revealed enveloped viruses with dumbbell-shape core (highlighted by white arrows) whereas in the ECTV-infected RAW cells, several immature virus particles were observed to accumulate in a large inclusion body. The inclusion body represents virus factory where intracellular mature virus assembles. This observation concords with the cytoplasmic peripheral staining observed in Figure 1C, suggesting that the RAW cells are permissive cells for poxvirus replication and suitable to study host response.

Collectively, the qPCR, immunofluorescence microscopy and ultrastructural observation indicated that the murine macrophage cell line, RAW cells are susceptible to infection by VACV, CPXV, and ECTV. Although our imaging analysis indicated different anti-VACV staining patterns, our analysis collectively indicated that RAW cells are suitable as an in vitro study model to investigate and compare the host response for the infection of poxvirus with different host range. Our analysis also indicated that a time point of infection between 10 to 24 hpi would be sufficient to study the virus-host response.

### 3.2. Global Host Cell Gene Expression in CPXV, ECTV, and VACV-Infected RAW Cells Showed Common and Virus-Specific Gene Expression Changes

The effect of global gene expression of the 3 poxviruses on infected RAW cells was examined using the GeneChip mouse genome 430 2.0 Array. RAW cells were infected with each poxvirus and at 2 and 16 hpi, the host transcriptome was analyzed. The 2 hpi time point corresponds to the early virus gene expression before the appearance of anti-VACV staining, while 16 hpi is at the time of infection before cell death commenced. Table 1 shows the overall summary of probe-sets showing up-regulated and down-regulated gene expression at 2 and 16 hpi, using a 2-FC cut-off. At 2 hpi, there were fewer probe-sets showing up-regulated gene expression at between 0.6% to 36% (CPXV-infected RAW cells at 36%; ECTV-infected RAW cells at 9.4%; VACV-infected RAW cells infected at 0.6%), and more probe-sets showing down-regulated gene expression at between 64% to 99.4% (CPXV-infected RAW cells at 64%; ECTV-infected RAW cells at 90.6%; VACV-infected RAW cells at 99.4%). At 16 hpi, the number of probe-sets showing up-regulated gene expression remained small, at between 4.1% to 18.3% (CPXV-infected RAW cells at 4.1%; ECTV-infected RAW cells at 18.3%; VACV-infected RAW cells at 4.4%). However, there was a significant change in probe-sets showing down-regulated gene expression observed for all 3 viruses, at between 81.7% to 95.9% (CPXV-infected RAW cells at 95.9%; ECTV-infected RAW cells at 81.7%; VACV-infected RAW cells at 95.6%). These data showed that infection with different poxvirus can cause different global host gene response, with the most changes observed at 16 hpi, which corresponds to the late gene expression in the poxvirus replication cycle.

**Figure 2:**
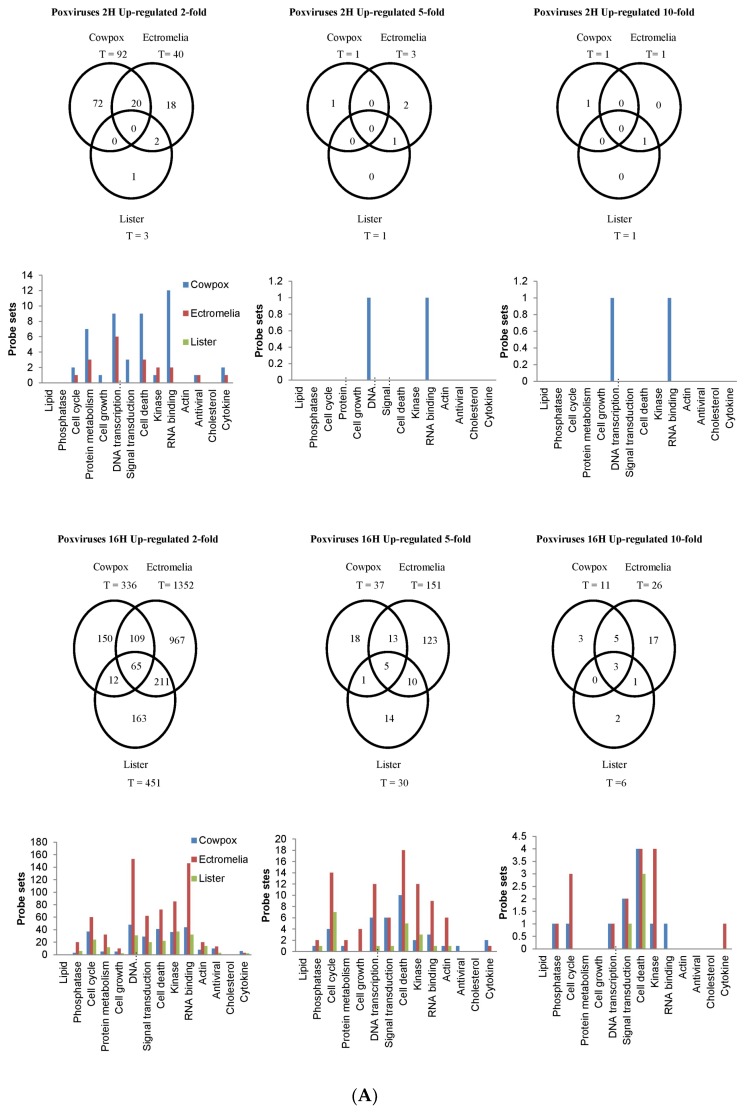

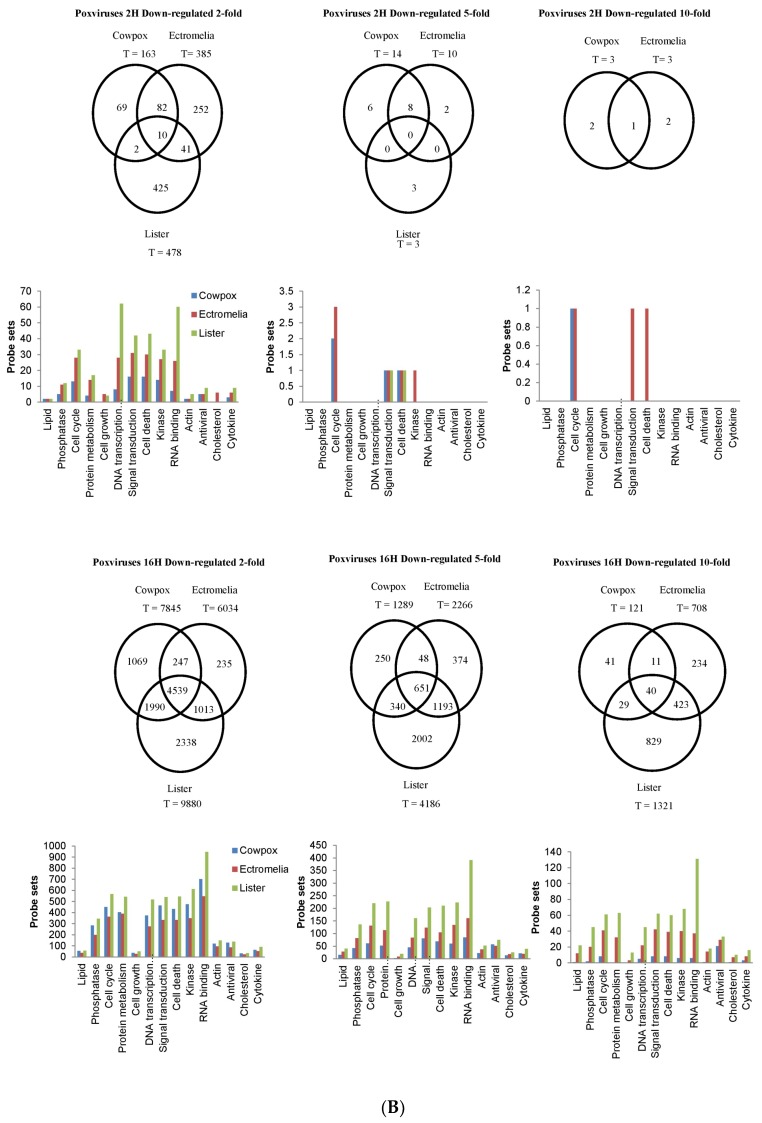
Temporal changes in global host cell gene expression in RAW cells infected with cowpox virus, ectromelia virus and vaccinia virus (Lister). The probe-sets showing significantly (**A**) up-regulated and (**B**) down-regulated gene expression at 2 h post-infection (2H) and 16 h post-infection (16H) are shown respectively in Venn diagrams and bar charts. In the bar chart, number of probe-sets are represented on the y-axis and the functional genes are represented in the x-axis. Changes in expression levels are based on 2, 5 and 10-fold changes (FC) (*p* ≤ 0.05).

The probe-sets showing up-regulated and down-regulated gene expression were filtered based on the FC exhibited (with respect to that in mock-infected cells). In this analysis we used a 2-FC, 5-FC and 10-FC cut-offs for differential expression and to distinguish probe-sets showing higher levels of change following virus infection. This also enabled us to compare these genes in cells infected with the 3 poxviruses and to identify genes that were common or unique to each poxvirus infection (Figure 2). The gene expression changes were compared with the mock-infected RAW cells, and with each poxvirus-infected RAW cells, and these FCs were represented in Venn diagrams and bar charts. At 2 hpi, there were no probe-sets showing up-regulated gene expression found common among the 3 viruses (Figure 2A). The highest changes in probe-sets showing up-regulated gene expression found in common between 2 poxviruses were between CPXV and ECTV-infected cells, and when filtered with 2-FC cut-off, was shown to be at 15.1% with probe-sets involved in DNA Transcription. As the infection progressed to 16 hpi, the probe-sets showing up-regulated gene expression using a 2-FC cut-off found common to the 3 viruses were at 3.0% and contained probe-sets from RNA binding, DNA Transcription, Kinase, Cell Death, Signal Transduction, and Cell Cycle. When filtered with the cut-offs at 5-FC and 10-FC, the probe-sets showing up-regulated gene expression from Cell Death continued to show the highest changes among the 3 viruses. At 16 hpi, the largest number of probe-sets showing up-regulated gene expression using a 2-FC cut-off were identified to be common between ECTV and VACV-infected cells and this was at 15.3%. At 2 hpi, the CPXV-infected cells showed the highest number of probe-sets showing up-regulated gene expression filtered at 2-FC cut-off that were unique to the virus, at 78.3%, and these probe-sets were mostly from RNA Binding and DNA Transcription. However, at 16 hpi ECTV-infected cells showed the highest number of probe-sets filtered at 2-FC cut-off unique to the virus at 71.5%, mostly in DNA Transcription and RNA Binding. When these were filtered further at 5-FC and 10-FC cut-offs, the unique probe-sets showing up-regulated gene expression from Kinase, Cell Cycle and Cytokine were observed in the ECTV-infected cells.

At 2 hpi, there were 1% down-regulated probe-sets using a 2-FC cut-off expressed in common among the 3 poxvirus-infected RAW cells and these were in RNA Binding, Cell Cycle, DNA Transcription, Signal Transduction, Cell Death, and Kinase (Figure 2B). When filtered at 5-FC cut-off, the probe-sets with the most changes were in Signal Transduction and Cell Death. Compared to 2 hpi, all 3 viruses generally exhibited higher changes in probe-sets showing down-regulated gene expression at 16 hpi with the 2-FC cut-off. The probe-sets identified in common among the 3 viruses were at 19.1% and were mostly in Phosphatase, Cell Cycle, Protein Metabolism, DNA Transcription, Signal Transduction, Cell Death, Kinase, and RNA Binding. At 16 hpi, the probe-sets using a 2-FC cut-off found in common between 2 poxviruses were between CPXV and VACV-infected RAW cells, and this was at 36.8%. At 2 hpi, the VACV-infected RAW cells showed the highest number of probe-sets with down-regulated gene expression unique to VACV. At 2-FC cut-off, this was shown to be at 89.0% and the probe-sets were found in DNA Transcription and RNA Binding. At 16 hpi, the VACV-infected RAW cells continued to show the highest number of probe-sets with down-regulated gene expression with 2-FC cut-off at 23.7% in RNA Binding. When filtered with 5-FC and 10-FC cut-offs, unique probe-sets mostly in RNA Binding were observed. The results showed here suggested that there were probe-sets representing virus-specific changes in the differentially expressed genes at both time points, as well as probe-sets found in common to all 3 viruses with more changes in down-regulated gene expression detected at 16 hpi.

### 3.3. CPXV, ECTV and VACV-Infected RAW Cells Induced a Range of Metabolism and Signaling Pathways

**Figure 3:**
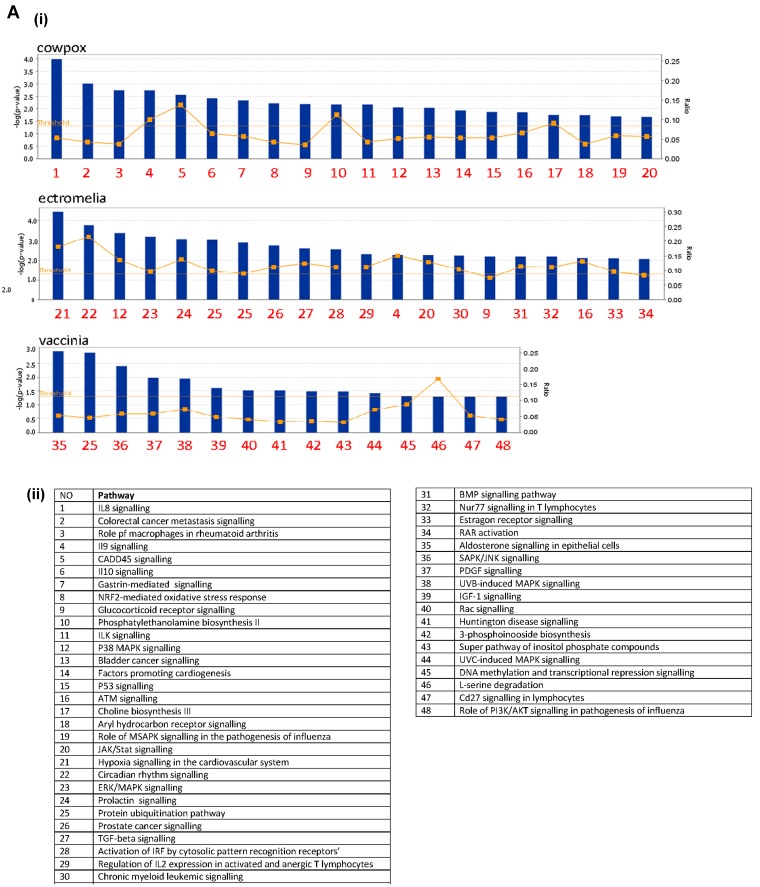

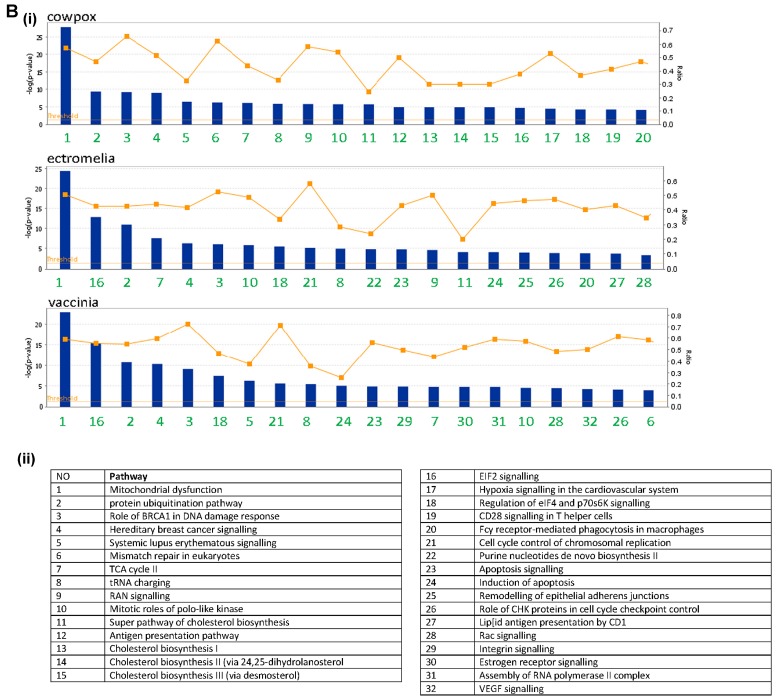
Different canonical pathways in RAW cells infected with cowpox virus (CPXV), ectromelia virus (ECTV) and vaccinia virus (VACV) showing differential gene expression. (**A**) Up-regulated and (**B**) down-regulated gene expression at 16 h post-infection (H). The microarray data set was processed using GeneSpring GX 11.0 and uploaded into Ingenuity Pathway Analysis version 2012 (IPA). The data was filtered based on the significance fold change (FC) cut-off ≤ to 0.05 and FC cut-off ≥ 2. Genes were categorized using IPA, and *p*-values were calculated by Fisher’s exact test for each canonical pathway. Threshold was set at *p*-value = 0.05 and indicated as –log (*p*-value) on the y-axis. (i) The top 20 statistically significant canonical pathways were identified for each infection time point and indicated by numbers on the X-axis. (ii) Table showing the numbers representing each pathways. The lines with orange boxes represent the ratio of the numbers of genes that show changes in gene expression data and the total number of genes in the respective canonical pathway.

Gene enrichment analysis was carried out using the ingenuity pathway analysis (IPA) for each virus infection time point, at 2 (Figure S2) and 16 hpi (Figure 3). The canonical pathways were identified at each time point and next ranked based on the corresponding p value (statistical significance calculated using Fisher’s exact test for each canonical pathway) using a cut-off of *p* < 0.05. The top 20 statistically significant pathways were identified and listed. For the up-regulated gene expression, more pathways were observed in each of the poxvirus-infected RAW cells at 16 hpi, and these were comprised of both metabolism and signaling pathways (Figure 3A). At this infection time point, there were more signaling pathways identified, and mitogen-activated protein kinase (MAPK) signaling pathways were observed in common among the 3 poxviruses-infected RAW cells, though they varied in their ranking. The metabolism pathways were more varied and unique for each poxvirus-infected RAW cells, compared to the signaling pathways. For the down-regulated gene expression, more canonical pathways were identified at 2 hpi, compared to the up-regulated gene expression at the same time point (Figure S2). Unlike the up-regulated gene expression, more metabolism pathways were observed at 16 hpi though these varied with each of the poxvirus-infected RAW cells (Figure 3B). Pathways representing the mitochondrial dysfunction, protein ubiquitination, and cholesterol biosynthesis were commonly identified among the 3 poxvirus-infected RAW cells. The number of genes showing changes in gene expression were next compared with the total number of genes showing changes in these canonical pathways and expressed in ratios. The canonical pathways for the up-regulated gene expression indicated ratios of between approximately 0.10 to 0.25 (i.e., between 10–25% of the total number of genes in the pathways). In contrast, the canonical pathways for the down-regulated gene expression indicated higher ratios of 0.10 to 0.8, suggesting that a larger number of genes were represented in these canonical pathways. Collectively, these data indicated that the poxviruses induced a range of metabolism and signaling pathways in RAW cells; with more response involving the down-regulated gene expression observed at 16 hpi.

### 3.4. CPXV, ECTV and VACV-Infected RAW Cells Showed Significant Down-Regulated Proinflammatory Cytokine Gene Expression Changes

**Figure 4:**
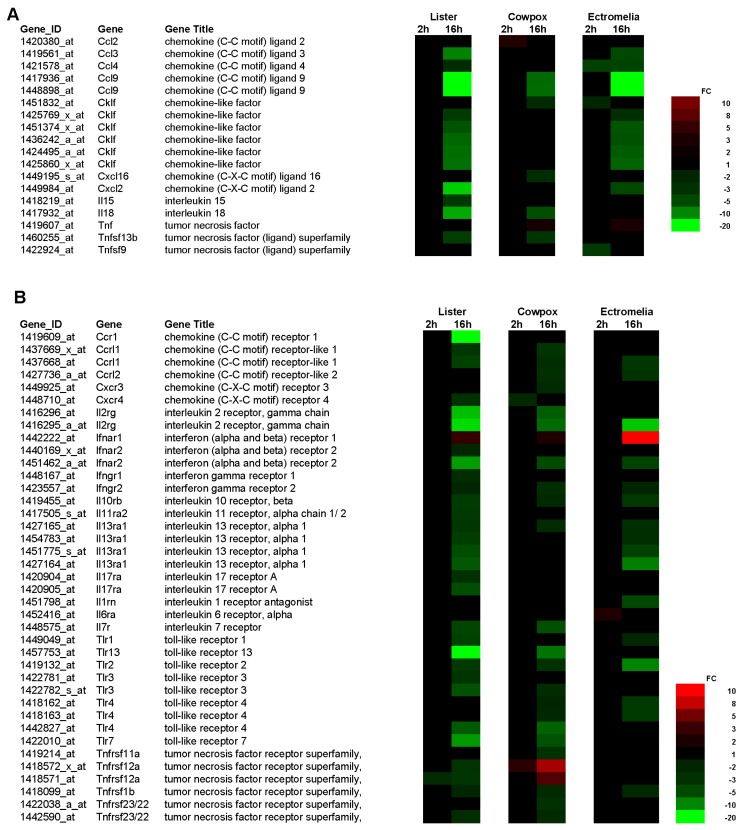
Changes in cytokine gene expression profiles in RAW cells infected with CPXV (cowpox), ECTV (ectromelia) and VACV (vaccinia virus; Lister). The fold change (FC) in cytokine gene expression involving cytokine ligands (**A**) and the cytokine receptors (**B**) at 2 and 16 h post infection (h) is presented as a heat map. The FC, gene identification (Gene ID), and gene name (Gene Title) are indicated.

The cytokine genes were examined in more details and selected genes representing ligands and receptors are shown as heatmaps in Figure 4A,B respectively. At 2 hpi, RAW cells infected with ECTV showed decreased expression changes in chemokine (C-C motif) ligand (Ccl) 4, chemokine-like factor (Cklf), tumor necrosis factor (ligand) superfamily (Tnfsf) 9, and increased expression changes in interleukin 6 receptor, alpha (Il6ra). At the same time point, the RAW cells infected with CPXV showed decreased gene expression in Ccl2, and chemokine (C-X-C) receptor (Cxcr) 4 but increased expression changes in tumor necrosis factor receptor superfamily (Tnfrsf)12a; and for RAW cells infected with VACV, only decreased gene expression change in Tnfrsf12a.

In contrast, at 16 hpi all 3 poxvirus-infected RAW cells showed decreased gene expression changes in both cytokine ligands and receptors. RAW cells infected with VACV showed the largest number of decreased cytokine gene expression changes as compared with RAW cells infected with either CPXV or ECTV. The cytokine genes with decreased expression changes found common to RAW cells infected with the 3 poxviruses were Ccl9, Cklf, chemokine (C-C motif) receptor–like (Ccrl) 1, interleukin 2 receptor gamma chain (Il2rg), interferon (alpha and beta) receptor (Ifnar) 2, interferon gamma receptor (Ifngr) 2, interleukin 10 receptor beta (II10rb), interleukin 13 receptor alpha 1 (II13ra1), toll-like receptor (Tlr) 2, Tlr4, and Tnfrsf1b. At 16 hpi, RAW cells infected with VACV and CPXV shared common decreased cytokine gene expressions in interleukin 18 (II18), Tnfsf13b, interleukin 7 receptor (II7r), Tlr3, Tlr4, Tlr7, Tlr13, and Tnfrsf23/22. Between VACV and ECTV, the infected RAW cells shared lesser common decreased cytokine gene expressions in Ccl3 and 4, Cxcl2 and Tlr1. There were also virus-specific cytokine genes showing decreased expression changes and in RAW cells infected with VACV, these were interleukin 15 (II15), Ccr1, Cxcr4, Ifngr1, interleukin 11 receptor alpha chain 2 (II11ra2), interleukin 17 receptor A (II17ra), and Tnfrsf12a. In the case of RAW cells infected with CPXV, there were fewer virus-specific cytokine genes with decreased expression changes and these were Cxcl16, Cxcr3 and Tnfrsf11a. The RAW cells infected with ECTV showed the least virus-specific cytokine gene with decreased expression gene changes in interleukin 1 receptor antagonist (ll1rn); and increased expression gene changes in Ifnar1.

These virus-induced changes were validated independently by measuring the mRNA level for each of the poxvirus-infected RAW cells at 2 and 16 hpi, using 2 types of PCR arrays, RT^2^ Profiler PCR Array Mouse inflammatory Cytokines and Receptors plates, and RT^2^ Profiler PCR Array Mouse Interferon and Receptors plates. As shown in Table 2, Ccl2 (−4.0 to −339 FC) and Ccl4 (1.2 to −29 FC) genes showed the most significantly decreased mRNA level for all 3 poxviruses-infected RAW cells at 16 hpi. The most significant increased mRNA level was observed for Il10 and for all 3 poxviruses-infected RAW cells. These changes in mRNA levels were between 4.9 to 12 FC at 2 hpi compared to between 11 to 30,806 FC at 16 hpi. The most significant increase in mRNA level was observed at 16 hpi for CPXV-infected RAW cells, with 30,806 FC. No changes in mRNAs were detected for Il2, Il5, Il12 and Il17 for both 2 and 16 hpi.

**Figure 5:**
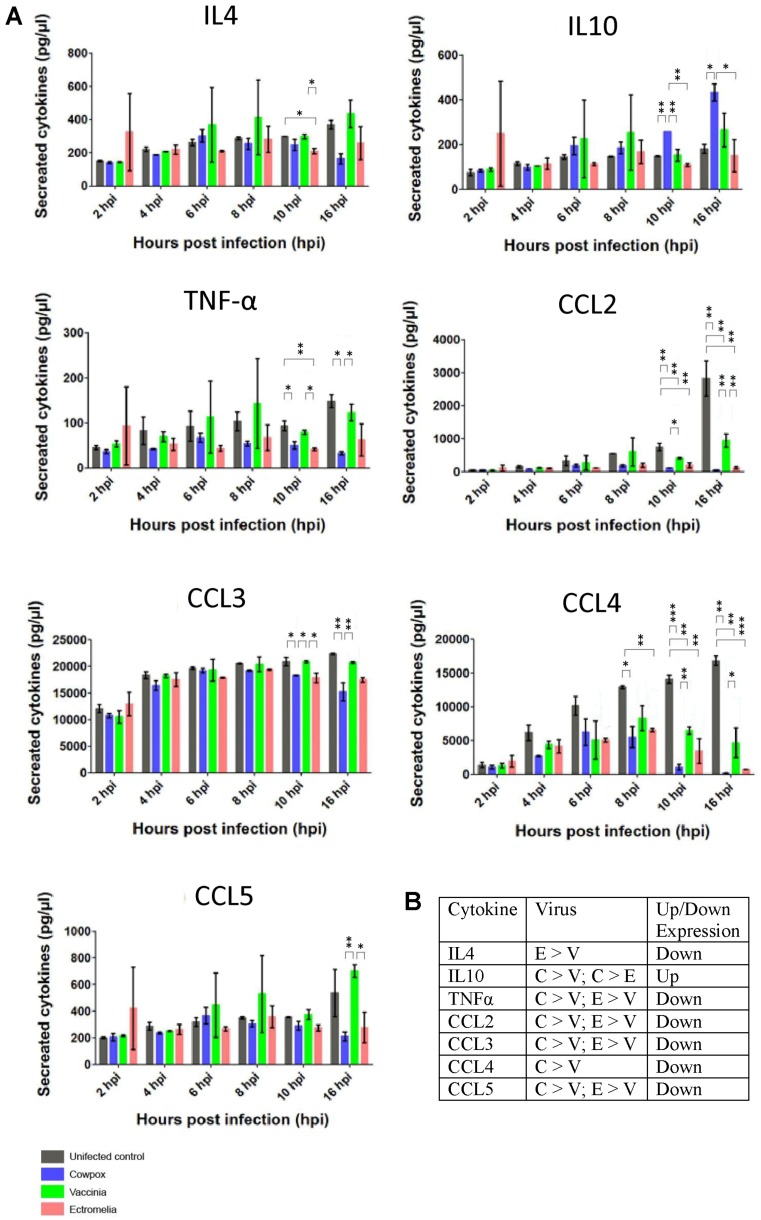
Detection of cytokines in the supernatant of RAW cells infected with cowpox virus, ectromelia virus and vaccinia virus. (**A**) From 2, 4, 6, 8, 10 and 16 h post-infection (hpi), the secreted cytokines were measured with the Bio-Plex Mouse Cytokine 23-Plex Panel (#M60009RDPD, Bio-Rad, Inc., Hercules, CA, USA). Data was analyzed using the Bio-Plex Manager software (Bio-Rad, Inc., Hercules, CA, USA) and pairwise analysis was performed with * representing *p* value < 0.05; ** < 0.01; and *** < 0.001. The amount of cytokines are indicated in pg/µL on Y-axis and time of infection as hpi is indicated on X-axis. (**B**) Table showing significant differences among the 3 viruses. C = cowpox virus, E = ectromelia virus and V = vaccinia virus. Up/Down indicates up-regulation or down-regulation of cytokine levels. Error bars are analysed from the average of 3 readings per sample.

In addition to the changes in mRNA we also measured the cytokine protein levels using the Bio-Plex Mouse Cytokine 23-Plex panel. In this way the levels of specific cytokines in the tissue culture supernatant of cells infected with the three poxviruses at between 2 to 16 hpi were measured. It would be expected that reduced mRNA levels would lead to either reduced protein levels of cytokines or no change in the cytokine levels when compared with that in mock-infected cells. This would depend on several factors e.g., the turnover rate of each specific cytokine protein. The most significant changes in cytokines were observed in Il4, Il10, TNF-α, Ccl2, Ccl3, Ccl4 and Ccl5 at 16 hpi for the 3 poxviruses-infected RAW cells (Figure 5A). No significant changes were observed for Il1, Il2, Il3, Il5, Il6, Il9, Il12, Il13, Il17, Ccl11, IFN-gamma, CxCl1, GM-CSF, and G-CSF (Figure S3). At 2 hpi, there was generally no difference in the cytokine expression between mock and VACV-infected RAW cells. Between 4 to 16 hpi, the most significant decreases in cytokines were detected for Ccl2 and Ccl4 in the supernatant of VACV-infected RAW cells when compared to the mock-infected RAW cells. Decreases in secreted cytokines for Il4, TNF-α, Ccl2, Ccl3 and Ccl4 were observed in CPXV-infected RAW cells at 16 hpi, except for Il10 which showed a significant increase. As early as 2 hpi, increases in cytokines expression were detected for Il4, Il10, TNF-α, Ccl5 in ECTV-infected RAW cells. At 16 hpi, these cytokines detected in ECTV-infected RAW cells showed a significant decrease in level. The results indicated that infection with each poxvirus in general induced no significant changes in the cytokine protein levels for 14 cytokines when compared with that in mock infections at 16 hpi. In specific cases significant reduction in cytokine protein levels was noted in 7 cytokines, namely in Il4, Il10, TNF-α, Ccl2, Ccl3, Ccl4 and Ccl5, with a significant increase in IL10. Although these general trends were observed for all viruses, the reduced cytokine protein levels were greater in CPXV-infected cells, and in the case of Il10, significantly higher (Figure 5B). Collectively the results suggested that gene expression involving anti-inflammatory cytokines are up-regulated and the pro-inflammatory cytokines, the chemokines, are generally down-regulated.

## 4. Discussion

Soon after the poxvirus enters the cell it is able to take over the host cell machinery, and reprogram the host cell to benefit its own viral life cycle to ensure a productive infection (see review by [18]). The point of entry for the poxviruses can be via the airway system, through the respiratory route, and in this context alveolar macrophages facilitate the spread of poxvirus within the host system [4,5]. The poxviruses are known to suppress the production of pro-inflammatory cytokines, so that the host innate immune response to the virus infection is dampened. This in turn affects the adaptive immune response and better understanding the mechanism of how the different poxvirus overcome these cellular defenses can derive novel virus interventions [19]. In addition, infected macrophages can contribute to virus dissemination within the host, and presumably poxviruses are able to impair anti-viral responses and enhance pro-viral responses in these cells [4,5]. The use of murine macrophage RAW cells to study virus-host response was reported with ECTV and myxoma virus, and several studies with DNA viruses [9,10,11,12,13,14]. Although a previous study had reported the use of RAW cell line to study antiviral activity of VACV, the study was rather limited and demonstrated only that Il15 stimulated the RAW cells to release nitric oxide, which inhibited VACV replication in a bystander human 293 cells [20].

Poxviruses enter host cells by fusion of the mature virions and cellular plasma membranes, releasing into the cytoplasm the virion core, from which early transcripts (synthesized within the core) are secreted. Upon subsequent uncoating, the released DNA genome is replicated, after which intermediate, then late, gene transcription can take place. The early genes encode the non-structural proteins including enzymes and other proteins required for the replication of virus DNA genome which can be detected as early as 2 h for VACV [21]. This would explain the observation that metabolism pathways were significantly down-regulated at 2 hpi during poxvirus infection of RAW cells (Figure 3). The poxvirus would be hijacking the host system and diverting cellular resources to viral replication. After virus replication, the late genes expressed the structural proteins to allow the formation of virus particles. At 16 hpi, we observed that the metabolism pathways in the infected RAW cells continued to down-regulate as the virus continues to shut the host system further down to permit efficient production of virus proteins for the formation of progeny viruses. Our results with the RAW cells infected with the 3 poxviruses further suggested that an infection time point at 16 hpi was adequate to study the global host response. Beyond 24 hpi, cell death commenced, suggesting that the optimal productive infection has completed with the release of infectious viruses, which rendered the study of host response impossible.

Our study showed that the production of pro-inflammatory cytokines was generally suppressed in infected RAW cells for all 3 poxviruses and this observation was reported as a common strategy deployed by poxviruses to evade the host innate immune system [19]. In our study, there was a significant down-regulation of host chemokine ligands, factors and receptors for each of the poxvirus-infected RAW cells at 16 hpi (Table 2; Figure 4 and Figure 5). The poxviruses are known to secrete viral homologues defined as viral chemokines Binding Protein (vCKBP) and these bind to chemokines secreted by host cells with high affinity, preventing the host chemokines from interacting with other inflammatory cells. Examples of vCKBP have been reported and these are A41 from VACV, and E163 and A41 orthologues from ECTV [22,23,24]. The results from our study suggested that the vCKBP are actively binding to the host chemokines secreted by all the 3 poxviruses-infected RAW cells to down-regulate the overall chemokine production. We next examined if similar effects of pro-inflammatory cytokines such as Il1, Il18, TNF and interferons that were reported to be suppressed during poxvirus infection [19] were also observed in the infected RAW cells. The heatmap in Figure 4B showed that at 16 hpi, the Il1m (interleukin 1 receptor antagonist) was down-regulated in RAW cells infected with ECTV, and this concords with the decrease of Il1a and b mRNAs (Table 2), and Il1 presence in the supernatant of infected RAW cells (Figure S3). There was also a decrease of Il1a and Il1b mRNAs for RAW cells infected with VACV, and only Il1a mRNA for RAW cells infected with CXPV at 2 and 16 hpi (Table 2). From the heatmap shown in Figure 4A, Il18 has the most decreased expression for VACV-infected RAW cells, followed by CPXV, and none detected for ECTV. Il12, which is associated with Il18, was suppressed in the supernatant of RAW cells infected with CPXV and ECTV at 16 hpi, but increased with VACV infection (Figure S3). Together with Il12, Il18 induces cell-mediated immunity and initiate production of gamma-interferon that continue to activate other macrophages. Poxviruses are known to encode viral homologues and in this case, Il18 binding proteins (BP) have been reported to bind directly to the pro-inflammatory cytokine Il18 with high affinity as a decoy receptor [25]. The Il18-BP interaction was important as it was reported to confer a virulence mechanism to VACV and CPXV during poxvirus infection. Our results suggested that the IL18-BPs from each poxvirus are actively binding to the Il18, but the interaction can exert different effects on each poxvirus-infected RAW cells. We next examined the effects on TNF and interferons in the poxvirus-infected RAW cells. The heat map in Figure 4 showed that gene expressions for TNF and interferons, including their receptors, were generally down-regulated for all 3 poxvirus-infected RAW cells at 16 hpi, except for Tnfrsf12a for CPXV-infected RAW cells. Similarly the mRNAs for TNF-α and cytokines were suppressed in the supernatant of RAW cells infected with the 3 poxviruses at the same infection time point. Poxviruses are known to encode another viral homologue during infection, the viral interferon α/β Binding Protein (vIFNα/βBP), which also functions as a virulent factor [26,27]. Our results suggested that the vIFNα/βBP were interacting with different receptors for different poxviruses and this would explain the general down-regulation of interferons and related genes. The mRNA level generally showed the same trend of decreased levels with the pro-inflammatory cytokines secreted in the tissue culture supernatants of poxvirus-infected RAW cells. The most significant increase observed was in the levels of both the mRNA and secreted Il10, an anti-inflammatory cytokine. At 16 hpi, we noted that in the case of Ccl2 and Ccl4, the reduced cytokine protein levels were greater in CPXV-infected cells. However, there seemed to be a baseline level of cytokine proteins present in RAW cells, which remained unaffected by poxvirus infection. These data suggested that there may be common evasion strategies by the poxviruses to overcome the host immune system.

Several studies have reported global gene expression profiling on virus-host response using microarray analyses on different VACV strains, CPXV and ECTV [28,29,30,31,32]. The closest cell model, which is representative of the first tier of host defense, is the monocyte-derived dendritic cells, where similar microarray studies were conducted with CPXV, Modified VACV Ankara (MVA), New York VACV (NYAC), and canarypox virus vector ALVAC [33,34,35]. Virus-host studies using RNA transcriptomic analysis was also reported with VACV in L929 cells and monocyte-derived dendritic cells from smallpox vaccines [8,36]. In addition, virus-host response using microarray analysis was studied for a range of poxviruses in in vivo animal models such as mouse and rhesus macaques [7,35]. The different VACV strains used in various studies were reported to give different conclusions to our findings. However, these studies were performed in permissive cells lines. For, example the VACV strains MVA, IHD, and the strain NYVAC, a derivative of the VACV Copenhagen strain, up-regulated cytokines during infection with HeLa cells, with more apoptosis genes reported for NYVAC infections [29,30,31,33]. These VACV strains were attenuated with the intention to be used as virus vectors or smallpox vaccine candidate and different immune regulatory virus genes seemed to prevent the host from activating the antiviral mechanisms. However, the innate immune gene expressions were generally down-regulated when transcriptomes were analyzed using ECTV-infected murine model and smallpox vaccines [7,36]. These results are consistent with our observations which showed that during poxvirus infection the pro-inflammatory cytokines were generally down-regulated in the infected RAW cells. A caveat to note in our study is that only one virus strain per virus species was examined, and these were laboratory-passaged strains. To qualify the above observations for each species in infected RAW cells, more strains, both laboratory-passaged and field isolates, would have to be examined and their gene expression profiles compared. The vCKBP, Il18BP and vIFNα/βBP can bind to their respective host cytokines to prevent the host cells from communicating with other inflammatory cells. The interaction of Il18BP was reported to reduce IFN-gamma induction, which in turn reduced the level of natural killer cell activation [25,37]. The vIFNα/βBP was reported to initiate the IFN type 1 pathway by preventing the IFN-induced antiviral state [26,27,38,39]. Collectively, these interactions with different viral homologues can effectively facilitate virus spread and this would explain the highly transmissibility nature of the poxvirus during infection. Our study successfully demonstrated that the RAW cells can be used as an effective in vitro model to study virus–host response for a range of poxviruses. For example, this cell model could be used to investigate the efficacy of new and better smallpox vaccine candidates by studying the gene expressions of cytokines involved in both the innate and cellular responses before deciding on efficacy trials in in vivo models.

## Figures and Tables

**Table 1:** Summary of the probe-sets showing up-regulated and down-regulated gene expression. At 2 and 16 h post infection, RAW cells were infected with cowpox virus (CPXV), vaccinia virus (VACV), and ectromelia virus (ECTV).

	Number of Probe-Sets Changed (≥ 2 fold)		
	Up	Down	Total
CPXV 2 hpi	92 (36%)	163 (64%)	255
CPXV 16 hpi	336 (4.1%)	7845 (95.9%)	8181
ECTV 2 hpi	40 (9.4%)	385 (90.6%)	425
ECTV 16 hpi	1352 (18.3%)	6034 (81.7%)	7386
VACV 2 hpi	3 (0.6%)	478 (99.4%)	481
VACV 16 hpi	451 (4.4%)	9880 (95.6%)	10,331

CPXV, VACV, and ECTV represent cowpox virus, vaccinia virus and ectromelia virus; hpi, hours per infection; probe-sets with up-regulated (Up) and down-regulated (Down) gene expression with respect to mock-infected cells are indicated; the total (Total) number of probe-sets showing up and down-regulated gene expression in each experimental condition is also shown. In parenthesis, the percentage represents the number of probe-sets with up or down-regulated gene expression over the total sum.

**Table 2:** Changes in Cytokine mRNA measured using PCR array in RAW cells infected with cowpox virus (CPXV), vaccinia virus (VACV), and ectromelia virus (ECTV).

Cytokine	CPXV-Infected RAW Cells		VACV-Infected RAW Cells		ECTV-Infected RAW Cells	
	2 hpi	16 hpi	2 hpi	16 hpi	2 hpi	16 hpi
IL-1a	−1.3	−1.9	−1.3	−5.5	−1.6	−15
IL-1b	−1.1	2.2	1.4	−5.8	1.0	−5.7
IL-3	6.7	2.9	2.0	2.3	1.6	−1.1
IL-4	3.6	−1.2	2.4	2.9	−2.0	1.3
IL-6	−2.5	34	2.2	2.6	−1.4	−1.3
IL-9	2.1	−4.8	1.7	−12	1.0	−8.2
IL-10	5.6	30,806	4.9	11	12	41
IL-13	3.7	−2.0	4.0	1.8	1.0	1.3
CCL11	2.3	5.7	2.6	1.0	−3.0	−5.5
CCL5	2.5	−1.9	−1.8	−2.3	6.1	−3.2
TNF-α	1.1	−1.0	−6.3	−5.1	−1.6	−3.4
IFN-g	6.0	3.2	1.2	2.2	1.9	2.5
CXCL1	3.1	−1.0	1.2	1.6	1.9	1.0
CCL2	2.0	−4.0	1.7	−73	1.6	−339
CCL3	1.1	−1.6	−1.1	−31	−1.2	−31
CCL4	−1.2	1.2	−1.2	−6.8	−3.0	−29
GM-CSF	1.7	6.4	−1.2	12	−2.0	1.3
G-CSF	1.8	2.7	1.3	−1.4	−5.1	3.1

CPXV, VACV and ECTV represents cowpox virus, vaccinia virus and ectromelia virus; hpi, hours per infection. IL represents interleukin; CCL, chemokine (C-C motif) ligand; TNF, tumor necrosis factor; IFN, interferon; CXCL, chemokine (C-X-C) ligand; GM-CSF, granulocyte macrophage colony-stimulating factor; G-CSF, granulocyte colony-stimulating factor.

## Supplementary Materials

The following are available online at http://www.mdpi.com/1999-4915/10/12/692/s1. Figure S1: Examination of RAW cells infected with cowpox virus (CPX), ectromelia virus (ECTV) and vaccinia virus (VACC) using bright-field microscopy and transmission electron microscopy (TEM). Figure S2: Different canonical pathways in RAW cells infected with cowpox virus, ectromelia virus and vaccinia virus (Lister) showing differential gene expression at 2 h post-infection (2h). Figure S3: Detection of cytokines in the supernatant of RAW cells infected with cowpox virus, ectromelia virus and vaccinia virus.
