# Supplementary material for: A System Based-Approach to Examine Cytokine Response in Poxvirus-Infected Macrophages
Source: Viruses. 2018 Dec 5;10(12):692. doi: 10.3390/v10120692 (PMC6316232; doi:10.3390/v10120692)
Supplement: Supplementary file 1 [file viruses-10-00692-s001.zip › supplementary material/captions.docx]

**Supplementary Material**

**Figure S1.** Examination of RAW cells infected with cowpox virus (CPX), ectromelia virus (ECTV) and vaccinia virus (VACC) using bright-field microscopy and transmission electron microscopy (TEM). RAW cells were (A) mock-infected, (B) CPX-infected at 48 h post-infection (hpi), (C) ECTV-infected at 24 and 48 hpi, and (D) VACC-infected at 24 hpi and 48 hpi, and examined using bright field microscopy. (objective x10). (E) RAW cells were infected with CPX, ECTV and VACC at MOI=10 and at 24 hpi, scraped and pelleted. The infected cell pellets were embedded with the resin mixture (26% *v/v* Epon 812, 58% *v/v* Dodecenyl succinic anhydride) and 16% *v/v* Araldite 502. When polymerized, each infected cell pellet were ultra-sectioned, double-stained with lead citrate and uranyl acetate, and examined with TEM (Model 1010, Jeol_). The boxed areas and white arrows highlight the intracellular mature particles for CPX and VACC-virus infected RAW cells, and immature virions in ECTV-infected RAW cells. The bar scale denotes 0.2 µm.

**Figure S2.** Different canonical pathways in RAW cells infected with cowpox virus, ectromelia virus and vaccinia virus (Lister) showing differential gene expression at 2 h post-infection (2h). (A) Up-regulated and (B) down-regulated gene expression. The microarray data set was processed using GeneSpring GX 11.0 and uploaded into Ingenuity Pathway Analysis version 2012 (IPA). The data was filtered based on the significance fold change (FC) cut-off ≤ 0.05 and fold change cut-off ≥ 2. Genes were categorised using IPA, and p-values were calculated by Fisher’s exact test for each canonical pathway. Threshold was set at p-value = 0.05 and indicated as –log (p-value) on the y-axis.

**Figure S3.** Detection of cytokines in the supernatant of RAW cells infected with cowpox virus, ectromelia virus and vaccinia virus. From 2, 4, 6, 8, 10 and 16 h post-infection (hpi), the secreted cytokine were measured with the Bio-Plex Mouse Cytokine 23-Plex Panel (#M60009RDPD, Bio-Rad, USA). Data was analysed using the Bio-Plex Manager software (Bio-Rad, USA). The amount of cytokines detected in pg/µl are indicated on Y-axis and time of infection are indicated on X-axis. No statistically significant changes were detected in these cytokines.
