# Supplementary figures and images for: A System Based-Approach to Examine Cytokine Response in Poxvirus-Infected Macrophages
Source: Viruses. 2018 Dec 5;10(12):692. doi: 10.3390/v10120692 (PMC6316232; doi:10.3390/v10120692)

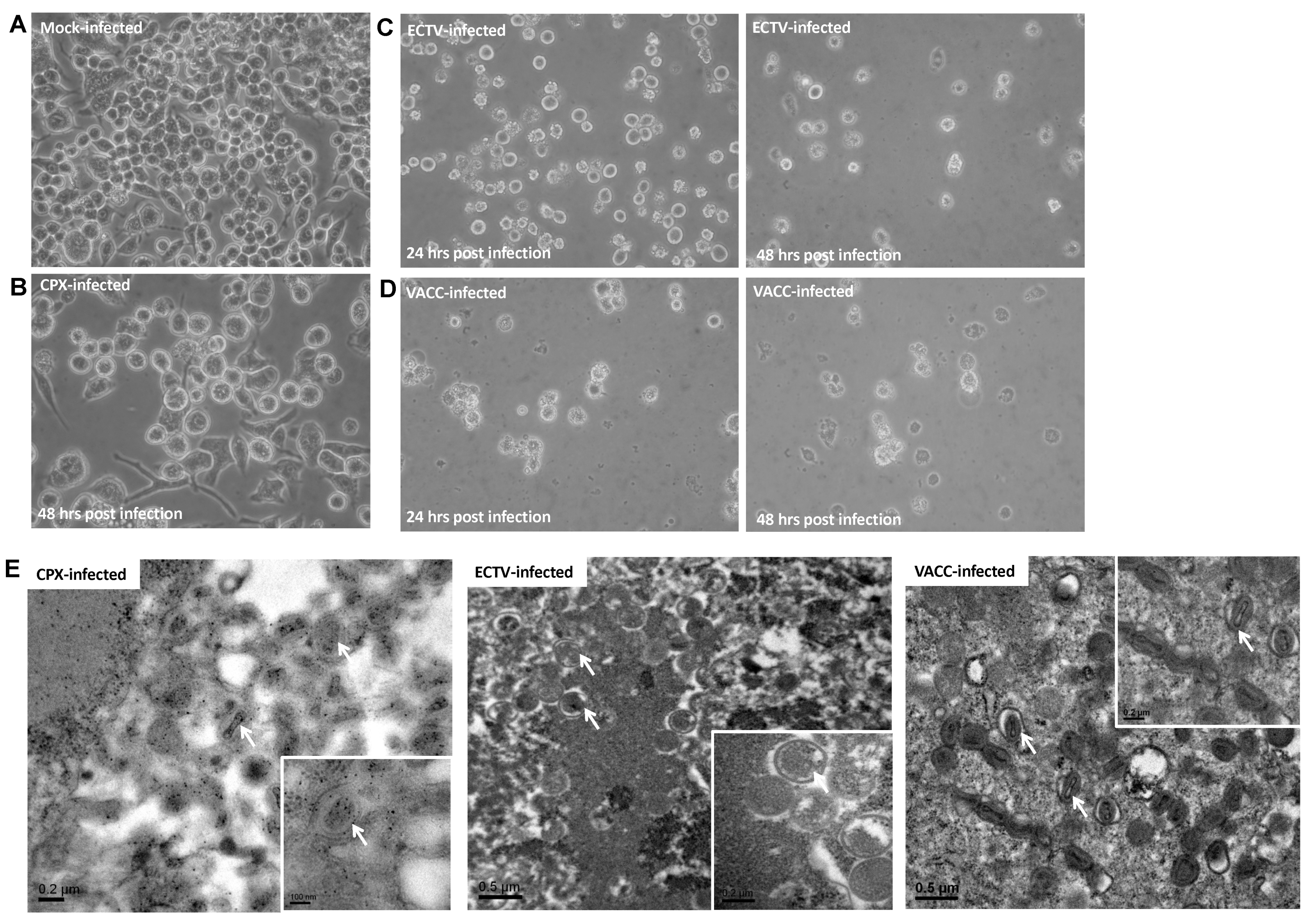

Supplement: Supplementary file 1 [file viruses-10-00692-s001.zip › supplementary material/SFigure 1.tiff]

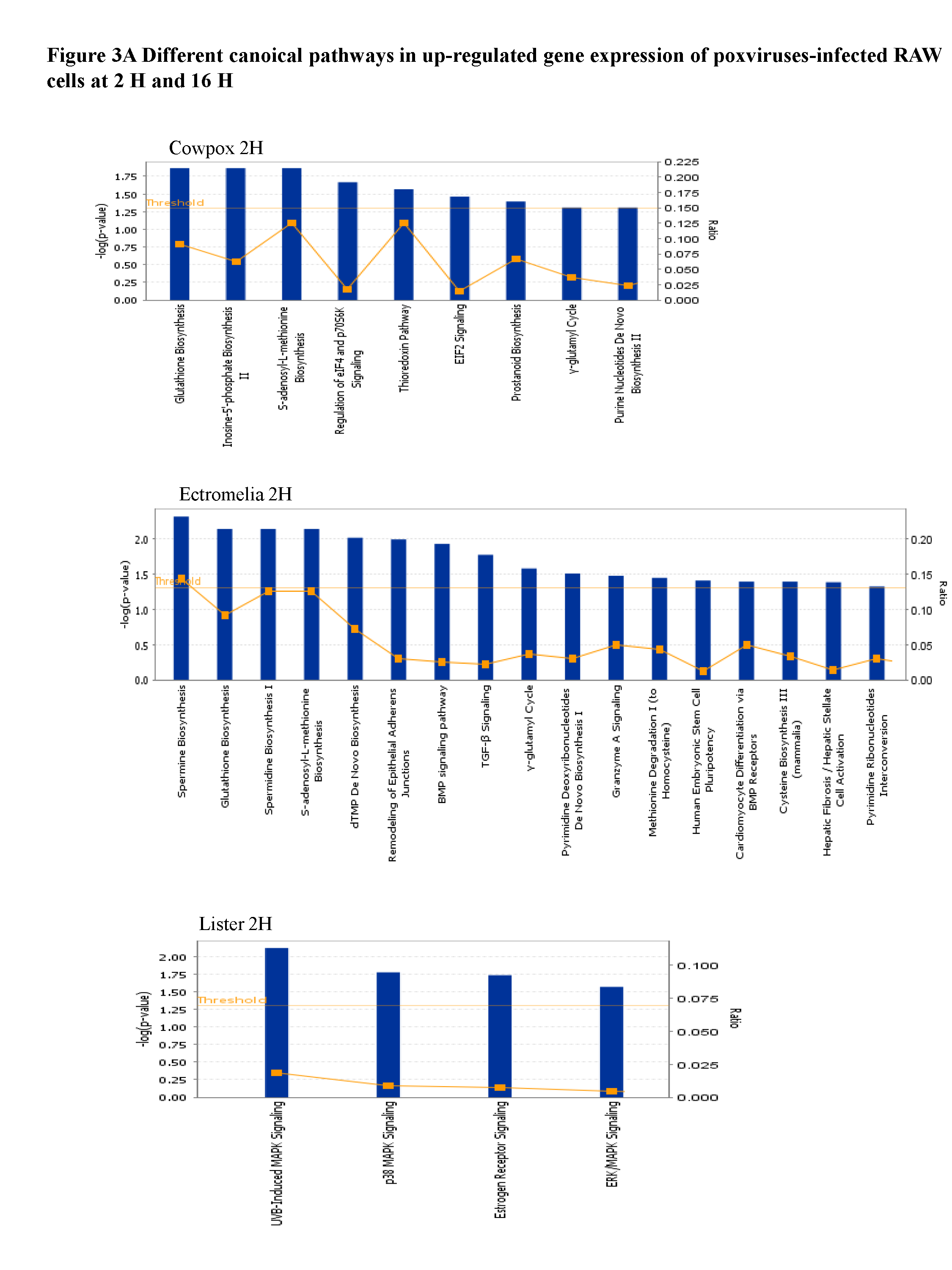

Supplement: Supplementary file 1 [file viruses-10-00692-s001.zip › supplementary material/SFigure 2A.tif]

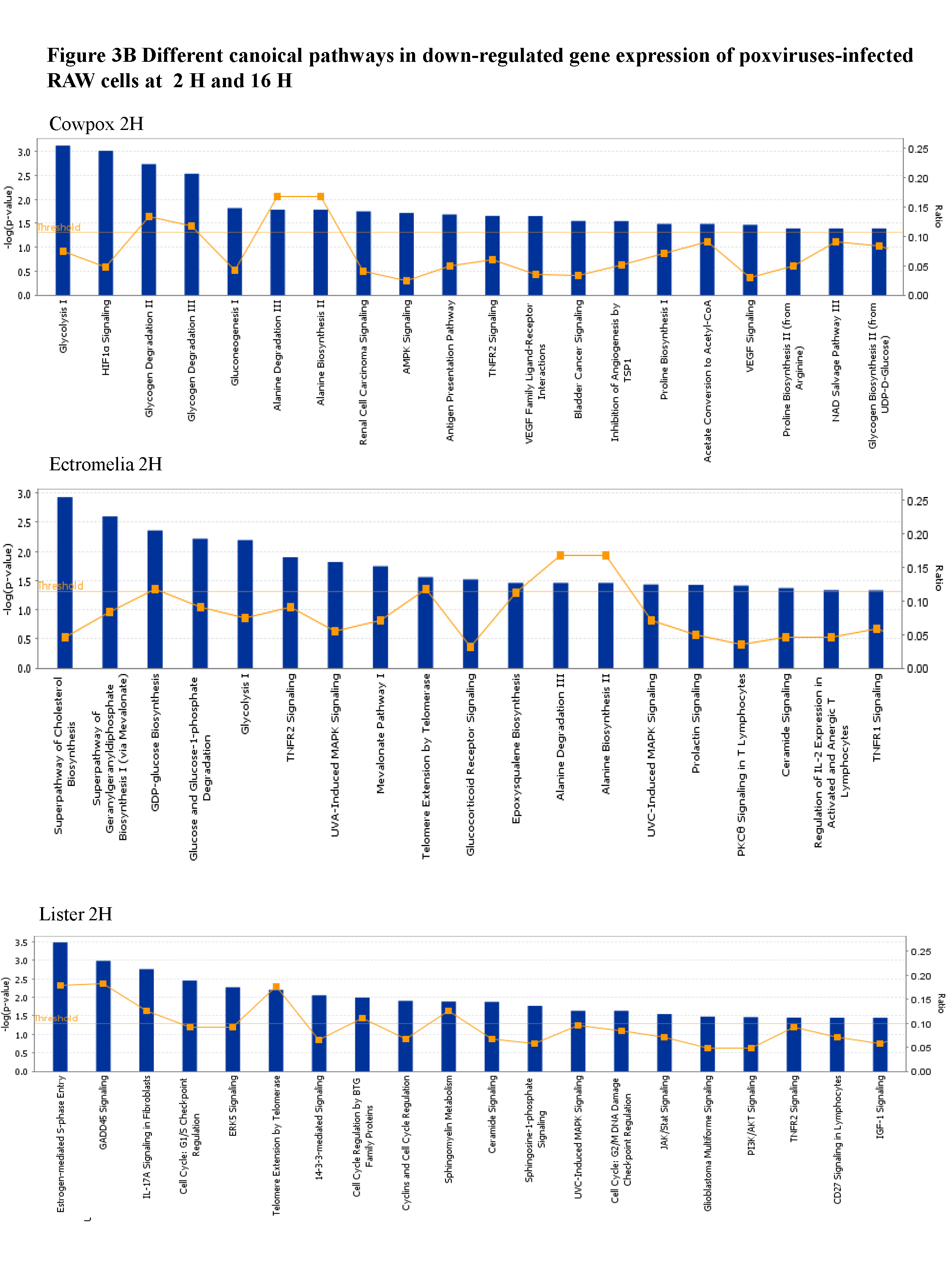

Supplement: Supplementary file 1 [file viruses-10-00692-s001.zip › supplementary material/SFigure 2B.tif]

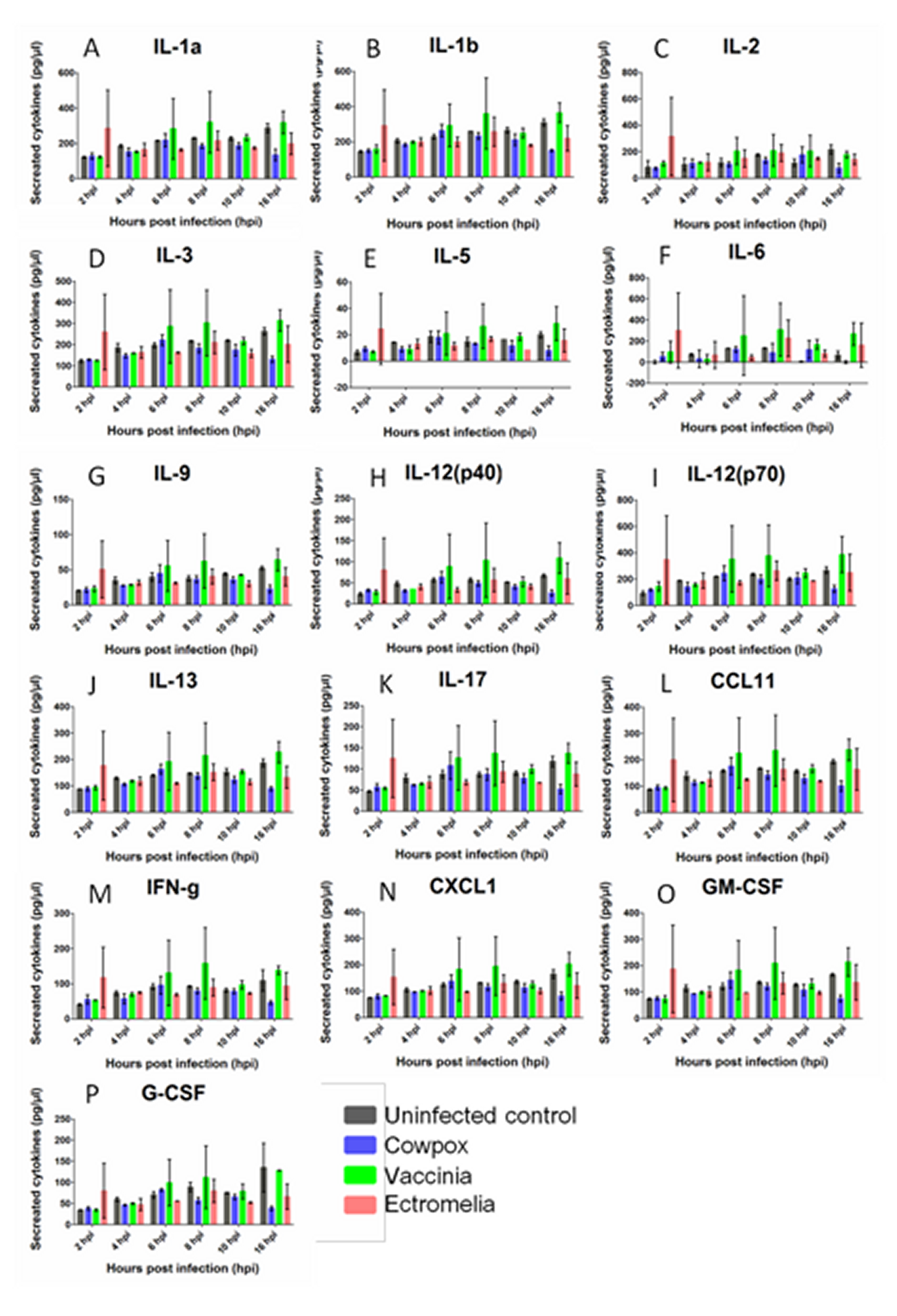

Supplement: Supplementary file 1 [file viruses-10-00692-s001.zip › supplementary material/SFigure 3.tif]
